# Draft Genome Sequence of Mycobacterium shimoidei Strain P7336

**DOI:** 10.1128/MRA.00957-18

**Published:** 2018-10-04

**Authors:** Jamal Saad, Anthony Levasseur, Michel Drancourt

**Affiliations:** a Aix-Marseille Université, IRD, MEPHI, IHU Méditerranée Infection, Marseille, France; Loyola University Chicago

## Abstract

Mycobacterium shimoidei, a slow-growing, nontuberculous mycobacterium initially isolated from a Japanese man, is a rare opportunistic lung pathogen. The 4,842,631-bp draft genome of M. shimoidei strain P7336 exhibits a 65.7% G+C content, 4,643 protein-coding genes, and 57 predicted RNA genes, including 49 tRNAs, 7 rRNA operons, and 1 transfer-messenger RNA.

## ANNOUNCEMENT

Mycobacterium shimoidei is a slow-growing, nontuberculous, and nonpigmented species sharing some phenotypic characters with M. malmoense and M. haemophilum (1, 2). Following its initial description as a lung pathogen in Japan (3), further cases have been described clinically that mimic pulmonary tuberculosis, with a thorax X ray usually presenting cavitation (4). These cases have been reported in Europe (4), Canada (5), Australia (6), and Madagascar (7). M. shimoidei has also been isolated from fish (8) and one diseased cat (9).

Here, we report the whole-genome sequence of M. shimoidei strain P7336. The isolate was cultured for 14 days on Middlebrook 7H10 agar supplemented with 10% oleic acid-albumin-dextrose-catalase (Becton, Dickinson, Sparks, USA) at 37°C under a 5% CO_2_ atmosphere. DNA was extracted using the InstaGene matrix (Bio-Rad, Marnes-la-Coquette, France), and 0.2 µg/µl of DNA was sequenced on the MiSeq platform (Illumina, Inc., San Diego, CA, USA). We made seven runs in a period of 39 h, with read lengths of 2 × 250 bp. Two libraries were prepared in the mate-paired format, one with inserts of 13 kb and one with inserts of 5 kb, and another library was prepared in the paired-end format. A total of 5,364,130 paired-end reads, filtered for quality, were assembled using SPAdes version 3.10.1 (10). To run SPAdes, we used the pipeline option “–careful” in order to reduce the number of mismatches and short indels. Default parameters for K values, i.e., *k*-mer values of 127, 99, 77, 55, 33, and 21, were applied. Contigs were combined by using SSPACE (11) and GapFiller (12) with default parameters. Manual finishing was performed by using similarity searches based on BLAST searches, and synteny blocks were detected with progressiveMauve alignment algorithms included in the Mauve software (13).

The M. shimoidei strain P7336 genome was found to contain six scaffolds and eight contigs, including two gaps, for a total of 4,842,631 bp and a 65.7% G+C content (*N*_50_, 4,829,736 bp; coverage, 46×). Annotation using Prokka version 1.12 (14) yielded 4,643 protein-coding genes and 57 RNA genes, including 49 tRNAs, 7 rRNA operons, and 1 transfer-mRNA. Further, *in silico* DNA-DNA hybridization (DDH) (15) values with reference genomes presenting >97% 16S rRNA gene sequence identity with M. shimoidei strain P7336 were estimated with GGDC version 2.1 (16). M. shimoidei strain P7336 showed 95.9% identity with M. shimoidei strain DSM 44152 (17), 92.4% with M. shimoidei strain HMC_M2, 22.7% with M. malmoense strain E614 and M. xenopi strain DSM 43995 (17), 21.8% with M. szulgai strain DSM 44166 (17), 21.7% with M. angelicum strain DSM 45057 (18), 21.5% with M. avium subsp. paratuberculosis strain k10 (19), 21.4% with M. shinjukuense strain CCUG 53584 (18), M. alsense strain DSM 45230 (18), and M. intracellulare strain MOTT-64 (20), 21% with M. kansasii strain ATCC 12478 (21), and 20.6% with M. haemophilum strain DSM 44634. These data confirm that strain P7336 belongs to the species M. shimoidei and suggest that this species does not belong to any currently described complex in the Mycobacterium genus.

### Data availability.

The draft genome sequence of M. shimoidei strain P7336 has been deposited in GenBank under the accession number UEGW00000000. The raw reads are publicly accessible at http://www.mediterranee-infection.com/article.php?laref=1067.
